# Teratogenic Effects of Intravitreal Injection of Bevacizumab in a Pregnant Rat Model

**Published:** 2017

**Authors:** Shahram Bamdad, Mina Bamdad, Mahsa Khanlari, Yahya Daneshbod, Behzad Khademi

**Affiliations:** a *Department of ophthalmology, Shiraz University of Medical Sciences (SUMS), Shiraz, Iran. *; b *Department of Developmental Biology, Islamic Azad University, Shiraz, Iran. *; c *Shiraz Molecular Pathology Research Center, Dr Daneshbod Lab, Shiraz, Iran.*; d *Shiraz University of Medical Sciences (SUMS), Shiraz, Iran.*

**Keywords:** Animal Model, Anti VEGF, Avastin, Itravitreal Injection, Peregnancy, Rat, Teratogenicity

## Abstract

In this research study, to investigate teratogenic effect of intravitreal injection of bevacizumab in pregnant rat model, twenty seven female Wistar rats were inseminated. Pregnant rats were divided into 6 groups (three groups as case and three as control groups). Each case and control groups were divided according to the day of intravitreal injections (day 2, 10 and 18). Rats in the case groups received 4 µL intravitreal injection of bevacizumab and the control groups received the same volume of distilled water. The tail and umbilical cord length in case groups 1, 2, and 3 did not display any significant differences compared to their control groups. The fetal weight was significantly lower in the case groups 1 (p>0.001) and 2 (p>0.001) compared to their control groups. Furthermore, the placental weight was only lower in the case group 1 (p>0.001). Case group 2 had a shorter crown rump length in comparison with its control group (p=0.029). Morphological investigations showed two abnormal cases of gastroschisis in group 1 and a case of a cleft in the skull in one of the rats in case group 2. The results show that intravitreal bevacizumab has developmental effect when administered in the early stages of pregnancy; but it is safe when administered in the last week of pregnancy in rats.

## Introduction

Recently, with the advent of anti-vascular endothelial growth factor (anti-VEGF) drugs and their effects on retina related diseases including choroidal neovascularization, diabetic macular edema, *etc*., the use of these drugs has increased remarkably(1). 

Bevacizumab is an anti-VEGF antibody approved by Food and Drug Administration (FDA) as a complementary medicine for the treatment of colon cancer and has been tested for other forms of cancers(2-6). 

The antibody attaches to all isoforms of VEGF and inactivates them (7, 8).

The drug is also used in treatment of many conditions like diabetes related hypoxia(9) macular degeneration, vitreal hemorrhage(10), cases unresponsive to laser treatment, and severe and refractory macular edema(9). 

 An intraocular injection of this drug is also used for treatment of choroidal neovascularization (CNV), or abnormal growth of the choroid which is due to multiple reasons such as age related macular degeneration, cystoid macular edema with uveitis(11), retinal vein occlusion(12), and diabetic retinopathy (13).

Although these conditions mostly occur in older patients, abnormal growth of vessels or neovascularization of the choroid, uveitis, myopia, ocular histoplasmosis, and ocular toxoplasmosis may also occur in females during fertility ages (14).

In America Two to three percent of births have some sorts of congenital defect(15), among them, 10% of the cases are due to extrinsic factors such as medicines and chemical substances. 

The off-label use of bevacizumab in treatment of neovascularization of the retina and in exudative diseases has increased since 2005 and is now universally applied (16) Meanwhile, the teratogenic side effects related to the use of this drug have been limited to a few studies.

With regards to the importance of eye diseases and the increasing use of bevacizumab, we tried to study the teratogenic effects of this drug on pregnant rats which can be a starting point for future human testing.

## Materials and methods

*Animal Husbandry and Maintenance*

Twenty seven adult female and five adult male Wistar rats with an initial average body weight of 190 ± 5 g and 2-3 months of age were included in the study. The rats were held in polycarbonate plastic cages measuring 15 ×20× 40 cm, the cages floors were covered with sawdust. Six rats were placed in each cage .The cages were washed every two days by water and disinfectants and sawdust were replaced. 

Care and use of the laboratory animals were in accordance with NIH guidelines. The rats had free access to commercial chow and tap water. They were placed in a temperature-controlled room (23 ± 1 °C) which had a 12 h light/dark cycle (lights on at 07:00 am).

This study followed the FDA Good Laboratory Practice regulations (CFR Title 21, Part 58) and the International Conference on Harmonization guidelines for reproductive toxicology testing.

*Procedure and investigation*

The rats were allowed to acclimatize for one week before the beginning of the experiment, after that all the female and male rats were mated. To determine the female rats sexual cycles and to reassure that they were all in the same phase of the strouse cycle, vaginal smears were taken from each of the rats. In cases that the cycles were not coherent with the other rats, estrogen and progesterone were used to organize the rats cycles. After mating, vaginal smears were taken from all the adult female rats in order to determine their pregnancy status and in case of pregnancy, that day was considered as the first gestational day for further experimentings. 

The rats were then divided into six groups including three case groups and their corresponding control groups. In each case group, six rats and in each control group, three rats were experimented. The case and control groups were classified based on the day that they received their intravitreal injections. The rats in group 1 received their injections on day 2 (gestational day) and those in groups 2 and 3 received their injections on days 10 and 18 (drug administration on the first, second, and third week for the three groups, respectively). The control groups received an injection of distilled water instead of bevacizumab in exactly the same conditions as their related case groups.

In the case groups, the rats were first anesthetized with an intramuscular injection of 0.3 mg/mL ketamine hydrochloride and 0.1 mg/mL xylazine. In order to sterilize their eyes, a mixture of ophthalmic ciprofloxacin and Betadine was applied onto the eyes with an eye dropper.

The rats received an intravitreal injection of bevacizumab solution (Genentech, USA) with a dose of 4 µL and a concentration of 25 mg/mL from a 2 mm distance from the limbus. Each rat of the control groups had an injection of distilled water in the same conditions as their corresponding case groups. After the injection, the rats were transferred into their cages until day 19 of pregnancy.

The average pregnancy period of the rats was between 21 and 23 days. The rats were euthanized by CO_2_ inhalation on gestational day 19 and each fetus was then extracted.

The fetusesʹ tale length, crown rump length (CRL) and umbilical cord length and fetal and placental weights were measured. After measuring these variables, the fetuses were kept in 10% formalin for further evaluations. The fetuses were then evaluated morphologically for neural tube, cleft lip, and digital ray abnormalities. The heart, kidney, liver, and vertebrae of the rats were investigated for defects and, in cases that an anomaly was seen, an autopsy was sent to the histology and pathology laboratories.

*Statistical analysis*

The data were analyzed using the Statistical Package for Social Sciences (SPSS) software for windows, version 15 (SPSS Inc., Chicago, IL, USA). The independent T-test, ANOVA test and, for further comparison of findings between the groups, the Tukey post-hoc test was used. All the data here are displayed as frequency and mean ± standard deviation (SD).

A p-value of ≤ 0.05 was considered as statistically significant.

## Results

The case groups 1, 2, and 3 each included 52, 52, and 44 rats, respectively, and the control groups 1, 2 and 3 each included 36, 36, and 4 rats, respectively. 

Regarding the effects of the intravitreal injection of the drug on the length of the rats tails in the three groups, our results showed that none of the rats in the case groups had a significant difference compared to their control groups (p = 0.23, p = 0.08 and p = 0.053 for groups 1, 2 and 3, respectively).

Regarding the CRL, no significant difference was seen between groups 1 and 3 in comparison with their corresponding control groups (p = 0.09 and p = 0.56 for groups 1 and 3, respectively). Case group 2 showed a significantly shorter CRL than its control group (3.9 ± 0.51 cm vs. 4.1 ± 0.2 cm; p = 0.029).

 The umbilical cord length did not display a significant difference between the groups that received an injection of bevacizumab and their control groups (p = 0.54, p = 0.68 and p = 0.29 for group 1, 2 and 3, respectively).

**Figure 1:**
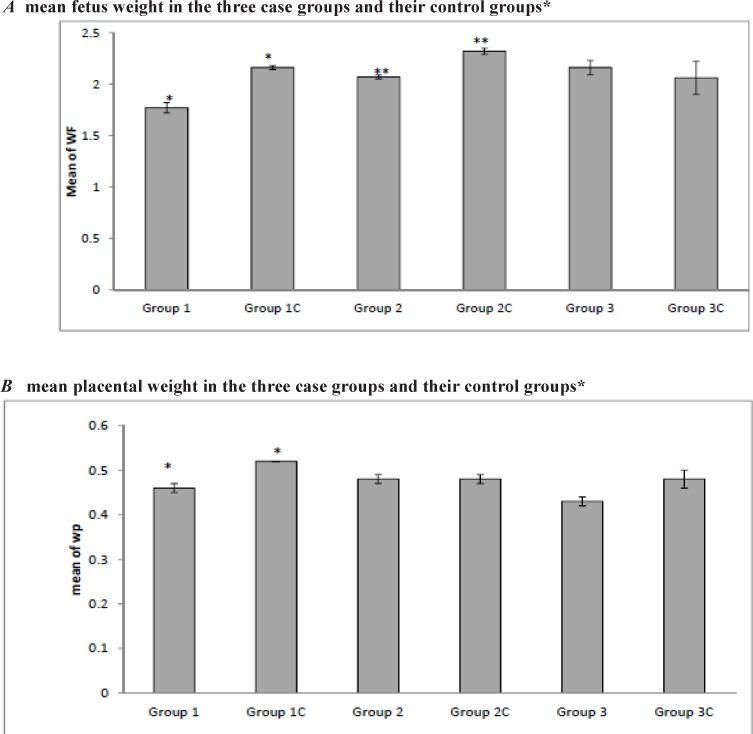
Fetal and placental weight in all the groups

After evaluating the effect of the drug on the fetal weight between the groups, our results showed that in case groups 1 and 2, a significantly lower birth weight was documented in comparison with their control groups (1.77 ± 0.37 g vs. 2.16 ± 0.12 g, p < 0.001; 1.96 ± 0.45 g vs. 2.32 ± 0.18 g, p < 0.001 for groups 1 and 2, respectively). This difference was not statistically significant for group 3 (p = 0.66). The placental weight was measured and our results showed that in case group 1 the placental weight was significantly lower than the control group (0.46 ± 0.07 g vs. 0.52 ± 0.05 g; p < 0.001). Regarding the placental weight, group 2 and 3 did not display a significant difference from their control groups (p = 0.87 and p = 0.19 for groups 2 and 3, respectively) (Figure 1 and Table 1).

The fetuses were evaluated morphologically. Our results showed that in case group 1, none of the rats had abnormal feet and hands, neural tube defects or cleft lip. Only two abnormal cases of gastroschisis were seen in group 1 presented with a liver protrusion. Furthermore, histopathological evaluations showed no microscopic abnormalities.

Morphological investigations in the case group 2 showed one case of cranial abnormality.

Neither the case nor control group 3 had abnormal findings in their morphological and microscopic investigations. Overall, none of the rats in the control groups displayed any morphological abnormalities. The fetuses were monitored and all still deaths were registered. Our results did not show any still deaths and abnormal bleeding in the total 222 fetuses that were studied. 

## Discussion

Bevacizumab administration did not affect the tail or umbilical cord lengths in the first, second, and third weeks of administration of the drug. So, based on our study, bevacizumab has no teratogenic effects on the mentioned parts. Our study showed that use of this drug in the first and second weeks led to a reduction in the fetal weight but this effect was not seen in the third week. The drug also affected the placental weight in the first week but did not have this effect in the second and third weeks. The drug affected the CRL in the rats which received the drug in the second week of gestation. So regarding the fetal weight, placenta weight and tail length, intravitreal use of bevacizumab in the third week of pregnancy is safe.

In a case report in 2010 (17), it was showed that intravitreal injection of bevacizumab in one eye reaches the other eye through the blood stream, and this entry in the blood provides a way for the drug to reach the fetus and cause subsequent anomalies. They concluded that anti-VEGF drugs inhibit the vascularization in the fetus and cause subsequent side effects, so the incidence of these anomalies depends on the time in which the drug is being used and should not be administered in the first trimester, when the vascularization process is most susceptible. This finding was similar to our results; as the administration of bevacizumab in the first week caused a decrease in the fetal and also, placental weight and injection in the second week caused a decrease in the fetal weight. The previous study concluded that this effect could be due to the drug affecting the vascularization in both the fetus and the placenta in the first week and the placental vascularization alone in the second week.

Diagram A shows that case group 1 and 2 both have a significantly lower weight in comparison to their control groups, yet this difference is not seen between case group 3 and its control group.

Diagram B shows that only case group 1 has a significantly lower placental weight in comparison to its control group, and this difference is not seen between case group 2 and 3 and their control groups. *Error bars represent 95% confidence interval.

**Table 1:** Comparison of case and control group.*

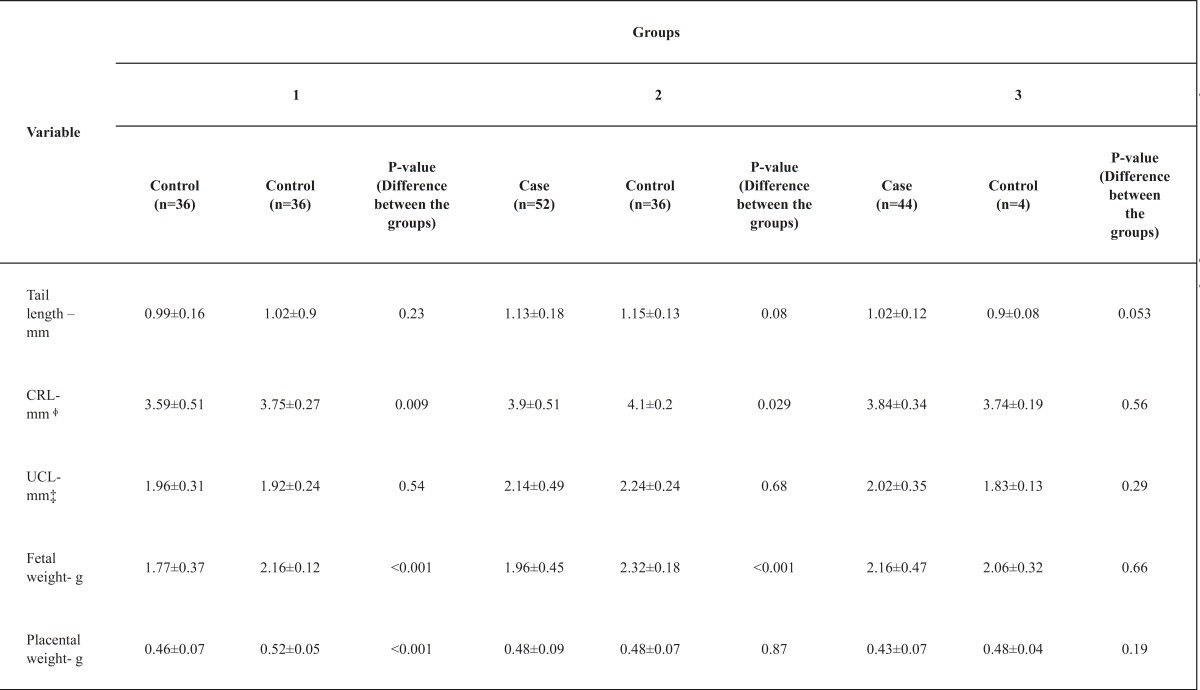

* All the plus- minus values are means ± standard deviation.

ᶲ CRL= Crown rump length.

‡ UCL= Umbilical cord length.

Other studies have evaluated other anti-VEGF drugs and have documented similar results. For instance, in a study by Patyna *et al*. (18), the effects of sunitinib malate was evaluated on the embryo–fetal development in rats and rabbits. They found that the drug caused skeletal abnormalities and decreased fetal weight.

In two other studies by Bakri *et al*. (19) and Rosenfeld et al (20), a 0.5 mg injection of *Ranibizumab* in rabbits, at the time that vascularization was occurring, resulted in an anomaly. 

Studies that have shown the effect of the drug on pregnant women have been mostly case reports or case series. In a case series by Tarantola *et al.* (14), the clinical course of intravitreal injection of Avastin was assessed in four pregnant women who suffered from choroidal neovascularization. The patients were followed up for one year and at the end, they showed no teratogenic effects. This finding was similar to the results documented in the rats that had bevacizumab injections in their third week of pregnancy. In the latter study, it was concluded that since bevacizumab was a large molecule, it could not pass the placenta and reach the fetus to cause anomalies. This conclusion was not in line with our findings since the use of the drug in the first week of pregnancy caused a decrease in the fetal weight. This inconsistency could be due to multiple reasons such as small sample size in the latter study or the differences in the placenta between humans and rats. 

The drug decreased the CRL in the rats in case group 2. This may need more investigation and studies due to our small sample size. 

In our study, the drug did not have any teratogenic effects on the fingers, neural tube, lips or the palate. Overall, three abnormalities were seen in the fetuses in the groups that received the injection in the first and second weeks, yet no microscopic abnormalities were documented in the fetuses. In the previously mentioned study conducted by Patyna *et al.* (18) the administration of sunitinib malate caused abnormalities in the chest wall, lumbar vertebrae, and a decrease in the fetal weight, unlike our findings that the anti-VEGF drug only caused a decrease in the fetal weight. This could be due to the differences in the characteristics of the two drugs.

Ferrara *et al*. (21) showed that a defect in the VEGF gene in the embryonic stem cells were lethal and in some cases caused anomalies in rats. In another study (22), the use of anti-VEGF drugs in pregnant rats inhibited growth in fetuses by disrupting the hormone production in the ovaries. In our study, we showed that the injection of the drug in the first weeks could not be safe and is probably safe when used in the last months of pregnancy. Unlike all the mentioned studies, some studies have found that the use of the drug has been associated with some devastating outcomes. In a study by Petrou *et al.* (23), two women who had intravitreal bevacizumab injections in the first days of pregnancy had early abortions. In the first case, a 29-year-old woman with sudden hemorrhage in the left eye who had associated diabetes; under control by insulin injections, had abortion 5 days after receiving bevacizumab. The second patient was a 25-year-old woman who was referred with decreased vision in her left eye. The patient had an abortion in her fourth week of pregnancy, ten days after receiving a dose of 1.25 mg bevacizumab. The same results were documented in the study by Gomez Ledesma *et al.* (24), where an intravitreal injection of bevacizumab for the treatment of CNV, caused by ocular histoplasmosis, led to a miscarriage. The authors linked this finding to impairment in the formation of blood vessels during the period of angiogenesis in the fetus. In our study, all the rats were alive and no cases of abortion were documented, but the impairment in angiogenesis that led to the reduction in fetal weight, was probably due to the effects of the drug. 

## Conclusion

Intravitreal injection of bevacizumab in Wistar rats decreases both fetal and placental weight when administered in the first week of pregnancy, and decreases the placental weight and crown rump length when injected in the second week. The drug does not cause any abnormalities in the microscopic findings. Regarding morphological anomalies, use of the drug in the first and second week of pregnancy can cause gastroschisis and cleft in the skull, respectively. Intravitreal injection of bevacizumab is safe when administered in the third week of pregnancy (third trimester) in rats.

## References

[1] Kourlas H, Schiller DS (2006). Pegaptanib sodium for the treatment of neovascular age-related macular degeneration: a review. Clin. Ther..

[2] Escudier B, Pluzanska A, Koralewski P, Ravaud A, Bracarda S, Szczylik C, Chevreau C, Filipek M, Melichar B, Bajetta E, Gorbunova V, Bay JO, Bodrogi I, Jagiello-Gruszfeld A, Moore N, AVOREN Trial investigators (2007). Bevacizumab plus interferon alfa-2a for treatment of metastatic renal cell carcinoma: a randomised, double-blind phase III trial. Lancet..

[3] Miller K, Wang M, Gralow J, Dickler M, Cobleigh M, Perez EA, Shenkier T, Cella D, Davidson NE (2007). Paclitaxel plus bevacizumab versus paclitaxel alone for metastatic breast cancer. N. Engl. J. Med..

[4] Sandler A, Gray R, Perry MC, Brahmer J, Schiller JH, Dowlati A, Dowlati A, Lilenbaum R, Johnson DH (2006). Paclitaxel-carboplatin alone or with bevacizumab for non-small-cell lung cancer. N. Engl. J. Med..

[5] Spaide RF, Laud K, Fine HF, Klancnik JM Jr, Meyerle CB, Yannuzzi LA, Sorenson J, Slakter J, Fisher YL, Cooney MJ (2006). Intravitreal bevacizumab treatment of choroidal neovascularization secondary to age-related macular degeneration. Retina..

[6] Vredenburgh JJ, Desjardins A, Herndon JE 2nd, Marcello J, Reardon DA, Quinn JA, Rich JN, Sathornsumetee S, Gururangan S, Sampson J, Wagner M, Bailey L, Bigner DD, Friedman AH, Friedman HS (2007). Bevacizumab plus irinotecan in recurrent glioblastoma multiforme. J. Clin. Oncol..

[7] Chiang CC, Chen WL, Lin JM, Tsai YY (2008). Effect of bevacizumab on human corneal endothelial cells: a six-month follow-up study. Am. J. Ophthalmol..

[8] Nyberg P, Xie L, Kalluri R (2005). Endogenous inhibitors of angiogenesis. Cancer Res..

[9] Chieh JJ, Roth DB, Liu M, Belmont J, Nelson M, Regillo C, Martidis A (2005). Intravitreal Injection of Triamcinolone Acetonide for Diabetic Macular Edema. Retina..

[10] Kook D, Wolf A, Kreutzer T, Neubauer A, Strauss R, Ulbig M, Kampik A, Haritoglou C (2008). Long-term effect of intravitreal bevacizumab (avastin) in patients with chronic diffuse diabetic macular edema. Retina..

[11] Adan A, Mateo C, Navarro R, Bitrian E, Casaroli-Marano RP (2007). Intravitreal bevacizumab (avastin) injection as primary treatment of inflammatory choroidal neovascularization. Retina..

[12] Prager F, Michels S, Kriechbaum K, Georgopoulos M, Funk M, Geitzenauer W, Polak K, Schmidt-Erfurth U (2009). Intravitreal bevacizumab (Avastin) for macular oedema secondary to retinal vein occlusion: 12-month results of a prospective clinical trial. Br. J. Ophthalmol..

[13] Rizzo S, Genovesi-Ebert F, Di Bartolo E, Vento A, Miniaci S, Williams G (2008). Injection of intravitreal bevacizumab (Avastin) as a preoperative adjunct before vitrectomy surgery in the treatment of severe proliferative diabetic retinopathy (PDR). Graefes Arch. Clin. Exp. Ophthalmol..

[14] Tarantola RM, Folk JC, Boldt HC, Mahajan VB (2010). Intravitreal bevacizumab during pregnancy. Retina..

[15] Hansen WF, Peacock AE, Yankowitz J (2002). Safe prescribing practices in pregnancy and lactation. J. Midwifery Womens Health..

[16] Grisanti S, Ziemssen F (2007). Bevacizumab: off-label use in ophthalmology. Indian J. Ophthalmol..

[17] Wu Z, Huang J, Sadda S (2010). Inadvertent use of bevacizumab to treat choroidal neovascularisation during pregnancy: a case report. Ann. Acad. Med. Singapore..

[18] Patyna S, Haznedar J, Morris D, Freshwater K, Peng G, Sukbuntherng J, Chmielewski G, Matsumoto D (2009). Evaluation of the safety and pharmacokinetics of the multi-targeted receptor tyrosine kinase inhibitor sunitinib during embryo–fetal development in rats and rabbits. Birth. Defects Res. B Dev. Reprod. Toxicol..

[19] Bakri SJ, Snyder MR, Reid JM, Pulido JS, Ezzat MK, Singh RJ (2007). Pharmacokinetics of intravitreal ranibizumab (Lucentis). Ophthalmol..

[20] Rosenfeld PJ, Rich RM, Lalwani GA (2006). Ranibizumab: Phase III clinical trial results. Ophthalmol. Clin. North Am..

[21] Ferrara N, Carver-Moore K, Chen H, Dowd M, Lu L, OʹShea KS, Powell-Braxton L, Hillan KJ, Moore MW (1996). Heterozygous embryonic lethality induced by targeted inactivation of the VEGF gene. Nature..

[22] Pauli SA, Tang H, Wang J, Bohlen P, Posser R, Hartman T, Sauer MV, Kitajewski J, Zimmermann RC (2005). The vascular endothelial growth factor (VEGF)/VEGF receptor 2 pathway is critical for blood vessel survival in corpora lutea of pregnancy in the rodent. Endocrinology..

[23] Petrou P, Georgalas I, Giavaras G, Anastasiou E, Ntana Z, Petrou C (2010). Early loss of pregnancy after intravitreal bevacizumab injection. Acta Ophthalmol..

[24] Gomez Ledesma I, de Santiago Rodriguez MA, Follana Neira I, Leon Garrigosa F (2012). Neovascular membrane and pregnancy Treatment with bevacizumab. Arch. Soc. Esp. Oftalmol..

